# Exploring Temporal Asymmetry in Human Behavior in Social Media Platforms

**DOI:** 10.3390/e28010027

**Published:** 2025-12-25

**Authors:** Liang Chen, Heng Zheng, Wenyue Wei, Haoran Pan, Weipeng Nie, Zhifeng Hao, Zhidan Zhao

**Affiliations:** 1 Department of Computer Science and Technology, College of Mathematics and Computer, Shantou University, Shantou 515063, China; chenliang@stu.edu.cn (L.C.); 24hzheng@stu.edu.cn (H.Z.); 13263889722@163.com (W.W.); hr.pan.kenny@gmail.com (H.P.); haozhifeng@stu.edu.cn (Z.H.); 2 School of Systems Science, Beijing Jiaotong University, Beijing 100044, China; wpnie@bjtu.edu.cn; 3 Department of BigData, School of Cyberspace Security, Hainan University, Haikou 570228, China; 4 Complexity Computation Laboratory, Hainan University, Haikou 570228, China

**Keywords:** critical time, human behavior, asymmetry, social media

## Abstract

With the continuous advancement of information technology, there has been a growing interest in understanding the complexities of human behavior. In particular, asymmetry in human behavior has emerged as a topic of significant theoretical and practical importance. This study focuses on investigating asymmetries in the context of critical time periods, an area that warrants immediate scholarly attention. To address this, we conduct a comprehensive analysis of datasets obtained from Douban movie reviews and Weibo comments related to a major product launch event, examining asymmetrical human activities surrounding pivotal temporal moments on these prominent social media platforms. In our exploration of behavior influenced by deadlines in social media, we propose a queue model that considers the interaction between neighbor effects and critical temporal frames in shaping individual task performance. Our results demonstrate the model’s effectiveness in accurately capturing observed patterns of temporal asymmetry, highlighting its potential as a valuable tool for understanding the impact of deadlines on human behavior in social media contexts.

## 1. Introduction

Asymmetry in human behavior, characterized by distinct patterns of actions and decisions in response to specific events or circumstances, is a well-documented phenomenon across various disciplines, including economics, psychology, and social sciences [1,2,3,4,5]. A notable aspect of human behavioral asymmetry is its temporal nature, where individuals exhibit different behaviors before and after critical events or deadlines [6,7]. For instance, individuals may procrastinate leading up to a deadline but become highly active and efficient as the deadline approaches.

The significance of deadlines in shaping human behavior has been extensively studied. Roth et al. observed the deadline effect in bargaining processes, where a significant portion of agreements were reached in the final moments before a deadline [8]. Duhigg’s work on “The Power of Habit” and Cox’s insights on leveraging deadlines further underscore the impact of deadlines on decision-making and behavior [9,10].

Moreover, the rise of social media has revolutionized human interactions and communication dynamics, playing a pivotal role in shaping real-world phenomena [11]. Social media platforms often witness surges in public discussions on specific topics, particularly around notable events or incidents [12,13,14]. This phenomenon, reminiscent of the avalanche effect in complex systems, highlights the rapid and sometimes unpredictable nature of information propagation on social media [15,16,17,18,19].

Despite extensive quantitative studies on criticality laws in social media information propagation, there remains a gap in understanding the dynamic processes underlying these phenomena. This paper addresses this gap by analyzing the causes and dynamics of critical phenomena from the perspective of human behavior. Leveraging empirical data from Douban movie reviews and Weibo comments on the Xiaomi SU7 car launch event, we examine the temporal statistical characteristics and their correlation with critical time.

In this study, we define critical time as the point at which individuals are prompted to complete tasks, emphasizing the importance of timely task completion. Building upon the task queue model proposed by Barabási and Albert [20] and integrating critical time effects and social influence, we develop a dynamic model to describe critical phenomena in social media information dissemination. Our numerical simulations validate the effectiveness of the model and explore the influence of different parameters on critical behavior dynamics.

## 2. Materials and Methods

### 2.1. Data Collection

The data utilized in this study were obtained from two primary sources: the Douban movie review dataset and the Weibo comment dataset. The Douban movie review dataset, sourced from the “Douban Movie Short Comments Dataset” on Kaggle (https://www.kaggle.com/datasets/utmhikari/doubanmovieshortcomments, accessed on 1 May 2025), comprises user comments regarding various movies available on the Douban website (https://movie.douban.com, accessed on 1 May 2025). On the other hand, the Weibo comment dataset encompasses comments extracted from Sina Weibo, focusing on discussions surrounding the 2024 Xiaomi SU7 car launch event.

To ensure the relevance and accuracy of the data, a rigorous selection process was employed. Specifically, data pertaining to a 30-day period before and after the critical event of interest were intercepted. This approach helped isolate and analyze data pertinent to the specific event under investigation while minimizing the influence of extraneous factors [21,22,23].

In this study, each user review of a film or event is treated as an individual event. The timing of user reviews corresponds to the activation period of the respective event within the temporal sequence. Critical points within the time series denote pivotal instances. Specifically, for the datasets analyzed in this paper, the “critical time” is defined as the film’s release date or the Xiaomi SU7’s launch date. These critical times serve as prompts for task completion, emphasizing the importance of engaging in activities around these specific junctures. Notably, completing tasks near critical times yields better outcomes compared to actions executed significantly distant from them [8,9,10,24].

To clarify, these two datasets were specifically selected because they both feature distinct and widely recognized critical time points—namely, the movie release dates and the product launch event. These moments serve as explicit “deadlines” that trigger concentrated user activity, making them ideal empirical cases for studying deadline-driven temporal asymmetry. By using these representative events, we aim to extract generalized patterns of human behavior that are applicable to various social media contexts centered around critical times.

### 2.2. Data Processing and Analysis

After collecting the data, several steps were undertaken to process and analyze it effectively. First, the data was cleaned to eliminate inconsistencies and irrelevant information: duplicate entries were removed; for missing data, the corresponding data entries were directly deleted; and timestamps were standardized by unifying their granularity to the daily level (i.e., only the year, month, and day information was retained).

Next, statistical analyses were conducted to identify patterns and trends within the datasets. Key metrics such as the frequency of user comments, the distribution of comments over time, and the relationship between comment activity and critical events were examined.

To further analyze the temporal dynamics of user behavior, the concept of critical timeframes was introduced. These critical timeframes represent periods of heightened user activity surrounding critical events. By analyzing the distribution of user comments relative to these critical timeframes, insights into the asymmetrical nature of human behavior were gained.

In Table 1, we present the basic statistics information of the data, including the name of the movie or event, the number of selected records, the total number of records, the interval in days between the peak time and the critical time *D*, and the frequency value of comments at the peak *P*. These statistics provide insights into the distribution and characteristics of user comments relative to critical events.

### 2.3. Model Development

Based on the insights gleaned from the data analysis, a computational model was developed to simulate the dynamics of critical phenomena in social media information dissemination. The model integrates critical time effects and social influence within a task queue framework, allowing for the exploration of various parameters and their impact on critical phenomena dynamics.

Numerical simulations were performed using the developed model to validate its efficacy and explore the influence of different parameters on the observed phenomena. Through these simulations, the model’s ability to capture the temporal patterns and asymmetries in human behavior was assessed, providing valuable insights into the underlying mechanisms driving critical phenomena in online social networks. Additionally, it is noteworthy that the model developed in this study to simulate the asymmetric effects of deadline behavior will be thoroughly described in the subsequent Section 3.

## 3. Results

In this section, we introduce our study’s central focus: pivotal statistics and asymmetry in human activity dynamics surrounding critical times. Our goal is to understand the rapid upsurge in human activity as critical times approach, while capturing its asymmetric nature. To achieve this, we employ a two-fold approach comprising empirical analysis and modeling. Through empirical analyses within social media platforms, we discern statistical regularities in human activity patterns around critical times. Subsequently, we develop statistical models aimed at replicating these observed patterns, particularly focusing on capturing their asymmetric features. Additionally, numerical simulations complement our empirical findings, enabling a deeper exploration of human activity dynamics surrounding critical times. Our simulation framework consists of two components: validating the efficacy of our statistical models and systematically exploring the influence of various parameters on model dynamics through controlled experiments.

### 3.1. Empirical Results

#### 3.1.1. Asymmetry in Human Activity Dynamics

**Figure 1:**
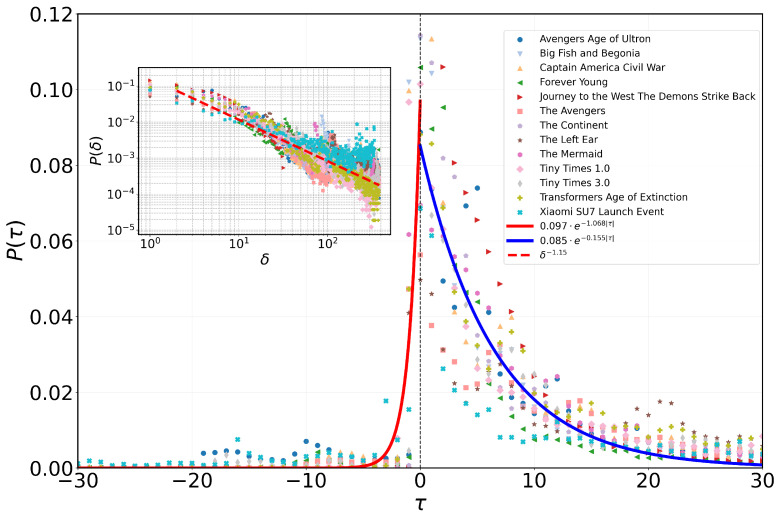
Distribution of Critical and Interval Times in Human Behavior. The main graphic depicts the time intervals between user-generated events of commenting behavior on various movies or product launch events and the peak of each event, denoted as τ. The frequency of these intervals is represented by the function P(τ). The curve enveloping the peak, P(τ), can be accurately described by the function $a\cdot e^{-b\left|\tau \right|}$. The fitting outcomes for both sides of the curve are manifested in the red and blue curves, corresponding to the left and right sides of the peak, respectively. Additionally, the inset chart illustrates the temporal disparity between event occurrences and their critical times, denoted as δ. On the double logarithmic axis, the distribution of δ approximates a linear trend, visually depicted as a red dashed line on the graph, with the slope corresponding to the power index of the distribution.

Our study commences with an examination of the temporal dynamics surrounding events, both preceding and following their zeniths. Figure 1 provides a visual representation of this analysis, depicting diverse movies and events with distinct colors and shapes. Here, τ denotes the time interval in days between event occurrence and the peak number of comments, with positive values indicating events occurring after the peak and negative values indicating events occurring before the peak. A consistent pattern emerges across various events, revealing a marked asymmetry in human activity dynamics surrounding critical times. Prior to the critical time, activity remains low, gradually intensifying as the critical time approaches. This intensification culminates in an exponential surge in user activity, peaking proximate to the critical time. Subsequently, there is a rapid decline in activity post-critical time passage. Importantly, these observations corroborate findings from prior studies on social network keywords, highlighting the prevalence of asymmetry in online human behavior during critical periods [12,25,26,27].

#### 3.1.2. Quantitative Analysis of Asymmetry

To quantitatively analyze this asymmetrical pattern, we perform a fitting analysis of the observed phenomenon. Our analysis reveals that the activity distribution preceding and succeeding the peak aligns accurately with an exponential function of the form $a\cdot e^{-b\left|\tau \right|}$, as this model can separately characterize the change patterns pre- and post-peak, inherently suiting the observed temporal asymmetry. We determine parameters $a_{1}$ and $b_{1}$ by fitting the left side of the peak and parameters $a_{2}$ and $b_{2}$ by fitting the right side of the peak. Specifically, for the left side, we ascertain $a_{1}=0.097$ and $b_{1}=1.068$, while for the right side, we find $a_{2}=0.085$ and $b_{2}=0.155$. The least squares method is employed for parameter fitting, optimizing parameter combinations to minimize discrepancies between fitting curves and empirical data. The computed coefficients of determination ($R^{2}$) are 0.985 for the left side of peak fitting and 0.971 for the right side of peak fitting, both close to 1, which attest to the quality of fit.

#### 3.1.3. Implications and Novel Research Avenues

The observed asymmetry in human activity dynamics during critical periods sheds light on emergent phenomena in user behavior scenarios, such as movies or major product releases. These phenomena reflect synchronized outbursts of substantial user engagement at pivotal junctures. Moreover, they underscore consistent underlying dynamics shared by diverse user activities during critical instants. The identification of asymmetry in human behavior during critical periods presents novel research avenues, including investigations into the onset of rare events, synchronized behaviors, and disease prevention and control [28].

#### 3.1.4. Understanding Critical Times in Human Behavior

Critical times play a pivotal role in shaping human behavior and have been extensively studied in the realm of human dynamics. Previous research has recognized deadlines as prominent examples of critical times [29]. Scholars have augmented task queue models with deadlines, elucidating the heterogeneity in human behavior [30]. In this study, we further explore the impact of critical times on event occurrence suddenness by focusing on temporal intervals between events and their respective critical times.

#### 3.1.5. Temporal Interval Distribution Analysis

We define the time interval δ as the absolute difference, in days, between a user’s behavior and the event’s critical time, irrespective of chronological order. Figure 1 illustrates the distribution of these time intervals δ for the behaviors under study. Notably, the outcomes highlight the heterogeneity within the distribution of time intervals near critical moments, aligning with the episodic nature of human behavior [31]. Remarkably, when δ falls between 2 and 383, the distribution conforms to a power-law distribution, passing the KS test with a power-law exponent of 1.15. This analysis, performed using the power-law package developed by Alstott [32], underscores the intrinsic heterogeneity within the time interval distribution and its profound influence on human behavior dynamics [20,33,34,35].

### 3.2. Model Description

**Figure 2:**
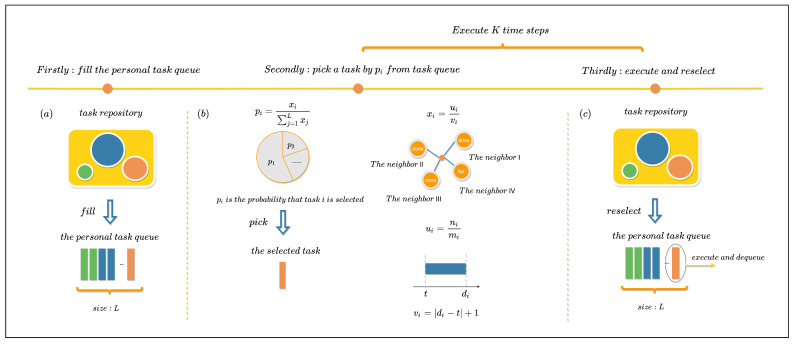
**Model Schematic Diagram.** The horizontal axis represents the step bar, with three dots indicating the three steps of the algorithm. (**a**) The initial step involves an individual extracting *L* tasks from the task repository to initialize their personal task queue. Tasks in the repository are illustrated as circles with varying colors and sizes, denoting distinct critical times. Yellow blocks within the individual’s task queue correspond to selected tasks, maintaining a queue length of *L*. (**b**) Task selection by the individual at each time step based on $p_{i}$. The pie chart below the $p_{i}$ formula illustrates the diverse probabilities associated with choosing each task ($p_{i},\;i=1,\ldots ,L$). The graph above the $a_{i}$ formula represents the individual and their neighbors, with task completion statuses displayed for task *i*. The number axis above the $b_{i}$ formula indicates the relative distance between *t* and $d_{i}$. (**c**) Executing the chosen task from the second step and appending a new task to the queue from the task repository. Steps two and three are iteratively repeated *K* times.

To deepen our understanding of the emergent phenomena related to the asymmetry of human behavior during critical moments, we propose a priority queue model that integrates social influence and critical time effects [1,36]. The model, depicted in Figure 2, accounts for the neighbor effect and the critical time of tasks on an individual’s task performance. It operates based on three key settings:

1. Each individual maintains a fixed task queue of size *L*.
2. A social network is established, where nodes represent individuals and connected nodes are neighbors.
3. Tasks are available in a communal task repository, with critical times following a distribution P(x).

The operational rules of the model are as follows:

1. Individuals randomly select *L* tasks from the task repository to form their initial to-do list.
2. At each time step, individuals stochastically choose a task from their queue for execution based on the task selection probability $p_{i},\;i=1,\ldots ,L$. This probability is dynamically adjusted according to the proximity of a task’s critical time to the present moment and the task’s execution status influenced by the individual’s neighbors.
3. Upon task completion, the algorithm removes the task from the individual’s queue and assigns a new task from the task repository, ensuring no overlap with existing tasks.

The task selection probability $p_{i}$ for tasks within the queue is calculated as:

(1) $$p_{i}=\frac{x_{i}}{\sum_{j=1}^{L}x_{j}}$$

where $x_{i}$ represents the priority value of task *i*, calculated by considering the neighbor effect ($u_{i}$) and the temporal disparity ($v_{i}$) between the current time step *t* and the critical time $d_{i}$ of task *i*:

(2) $$x_{i}=\frac{u_{i}}{v_{i}}$$

The neighbor effect $u_{i}$ is defined as the proportion of the individual’s neighbors who have completed task *i* among all the neighbors of that individual:

(3) $$u_{i}=\frac{n_{i}}{m_{i}}$$

where $n_{i}$ is the number of neighbors of the individual who have completed task *i*, and $m_{i}$ is the total number of neighbors for the individual. The temporal disparity $v_{i}$ is calculated as the absolute difference between the critical time $d_{i}$ and the current time step *t*, plus one:

(4) $$v_{i}=\left|d_{i}-t\right|+1$$

the core purpose of including the “+1” term is to avoid singularities: when the current time step *t* coincides exactly with the critical time $d_{i}$ of the task (i.e., $\left|d_{i}-t\right|=0$), this design prevents the denominator from being zero, ensuring the calculation of the task selection probability $p_{i}$ is mathematically valid and logically consistent.

The model operates within a complex network model *G*, where *N* tasks are introduced to the task repository at each time step *C*, with critical times generated from a uniform distribution. The model undergoes a total of *K* iterations. Table 2 summarizes the parameter values used in the model, which will be further discussed in the subsequent discussion section.

### 3.3. Numerical Results

To rigorously assess the effectiveness and robustness of our proposed model, we conduct a series of simulation experiments, systematically varying specific parameters while keeping all other parameters constant. This approach allows us to isolate the effects of individual parameters and evaluate their impact on the model’s performance. Our simulation study aims to validate whether the distributions of temporal distances τ between event occurrence time and peak time, and interval times δ between event occurrence time and critical time, closely resemble those observed in empirical data. Additionally, we investigate how specific parameters influence the distributions of τ and δ.

Specifically, we analyze the effects of the following parameters:

1. **Structure of the complex network (*G*):** The topology of the social network model plays a crucial role in shaping individual interactions and information propagation, thereby impacting task completion patterns.
2. **Length of the task queue (*L*):** The capacity of an individual’s task queue determines the number of tasks they can handle simultaneously, affecting their task selection strategies and overall productivity.
3. **Count of tasks (*N*) generated at discrete time *t*:** This parameter governs the rate at which new tasks are introduced to the task repository, influencing the overall task load and dynamics.
4. **Number of iterations (*K*):** This parameter defines the total duration of the simulation experiment and influences the convergence behavior and stability of the model dynamics.

Through comprehensive analysis and comparison with empirical observations, we aim to gain deeper insights into the behavior of our model under varying conditions and validate its suitability for capturing real-world phenomena.

#### 3.3.1. Effects of Network Structure *G*

Real-world networks exhibit diverse structural features, such as small-world effects and scale-free properties, distinguishing them from regular and random networks [37,38]. These characteristics, observed in small-world networks and scale-free networks, respectively, make complex networks valuable models for studying various real-world phenomena, including disease propagation, financial crises, and information dissemination [39,40,41,42].

To investigate the influence of network structure on our model, we employ two widely studied network models: the Barabási-Albert (BA) network and the Watts-Strogatz (WS) network. The BA network grows by preferential attachment, where new nodes tend to attach to existing nodes with higher degrees, leading to a scale-free topology. In contrast, the WS network exhibits small-world properties by rewiring a fraction of edges in a regular lattice, facilitating short path lengths and high clustering coefficients.

In our numerical simulations, we set parameters L=10, N=2L, C=30, and K=1000. For the WS network, we initialize a network with 4039 nodes, each connected to 44 neighboring nodes with a rewiring probability of 0.05. The BA network begins with $m_{0}=22$ nodes, each connected to 22 edges, and grows iteratively by adding nodes that preferentially attach to existing nodes based on their degrees, reaching a final size of 4039 nodes [32,37,38].

**Figure 3:**
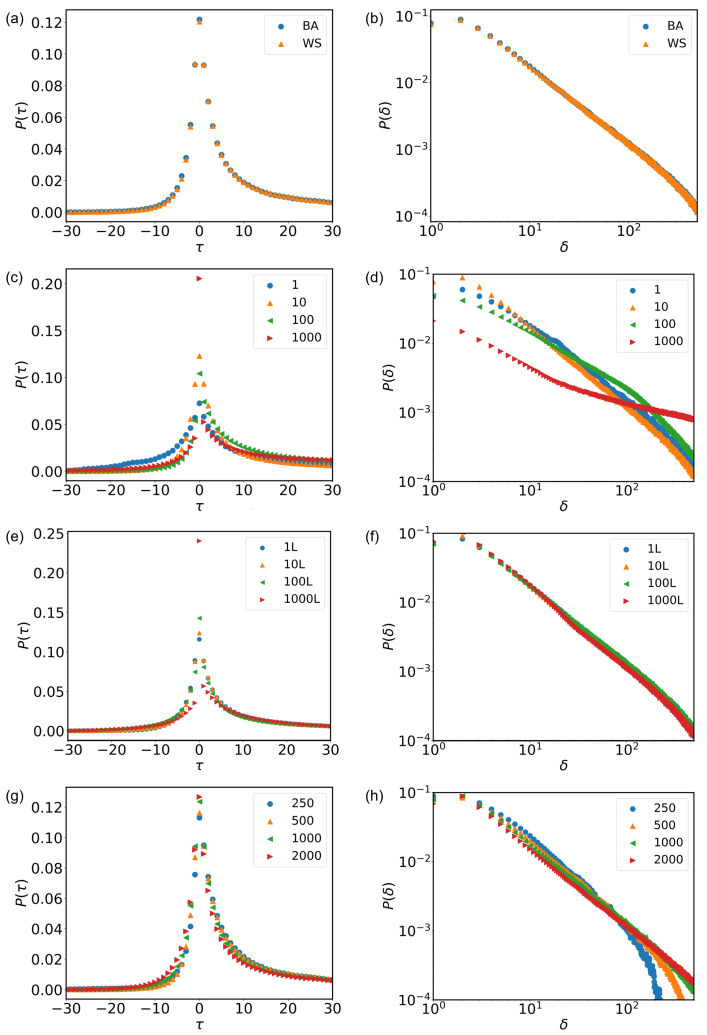
The Numerical Results. (**a**) Simulation of Complex Network Structure G’s Impact on Asymmetry Patterns: Analyzing Data Flow and Information Propagation. (**b**) Demonstrating the Effect of Complex Network Structure G on the Distribution of Interevent Times. (**c**) Impact of Queue Length L on Asymmetrical Behavioral Patterns in Network Systems. (**d**) Queue Length L and Its Influence on the Time Distribution of Event Occurrences. (**e**) Simulation of the Effect of Task Number N at Moment t on Network Behavior Asymmetry Under Varying Load Levels. (**f**) Task Number N at Moment t and Its Impact on the Distribution of Interevent Times. (**g**) Investigating the Influence of Iteration Count K on Asymmetry Patterns in Network Dynamics. (**h**) The Role of Iteration Count K in Altering the Distribution of Interevent Times.

Figure 3a illustrates the distribution of the time interval τ between event occurrence time and peak time, comparing simulations using the BA and WS networks. Notably, the BA network’s distribution exhibits a slightly more pronounced peak and marginally steeper decay, indicating faster ascent and descent phases compared to the WS network.

Figure 3b depicts the distribution of the time interval δ between the event occurrence time and the critical time, where both BA and WS networks exhibit power-law behavior on a logarithmic scale. Table 3 presents the fitting results of the observed power-law distributions in these simulations [32]. The power-law distribution of the BA network decays slightly faster, indicating that individuals in the BA network have higher reactivity to the critical time effect. This is attributed to the lower clustering coefficient of the network, suggesting that sparse connections lead to a greater impact of the critical time effect during the task selection process [32,37,38].

Overall, our numerical simulations demonstrate that network structure influences the dynamics of task completion and the responsiveness of individuals to critical time effects, reaffirming the validity of our model in capturing real-world phenomena.

#### 3.3.2. Impact of Task Queue Length *L*

The length of the task queue (*L*) plays a crucial role in shaping task execution dynamics and individuals’ responsiveness to critical time effects. A larger *L* allows individuals to maintain a longer list of pending tasks, which provides potential flexibility but also introduces a complex dual impact—enhanced temporal concentration and increased risk of extreme delays. In contrast, a smaller *L* restricts the number of pending tasks, typically exhibiting a behavioral pattern characterized by more dispersed task completion times but lower delay risks.

To investigate the influence of *L* on our model, I conducted numerical simulations by varying *L* while keeping other parameters constant. Specifically, we set N=2L, C=30, and K=1000. The simulation results reveal distinct patterns in the distributions of the time interval τ between event occurrence and peak time, as well as the time interval δ between event occurrence and the critical time, for different values of *L*.

Figure 3c illustrates the distribution of τ under different *L* values. As *L* increases, the τ distribution becomes noticeably sharper and more concentrated around τ=0. This indicates that the increase in task queue length *L* significantly enhances synchronicity in inter-individual task participation timing. Conversely, for smaller *L* values (1 and 10), the distributions are flatter and broader, indicating more dispersed task participation and weaker synchrony. These observations remain consistent with empirical findings, following an exponential pattern of $a\cdot e^{-b\left|\tau \right|}$. The exponential coefficients on both sides of the peak are shown in Table 3.

Similarly, Figure 3d presents, in log-log coordinates, the distribution of δ under different *L* values. As *L* increases, the decay characteristics change markedly. For smaller *L* values, P(δ) decays rapidly, suggesting a lower probability of long delays. However, as *L* increases, the heavy-tail feature of the distribution becomes more pronounced. This implies that when the task queue is longer, individuals are more likely to postpone task engagement far from the critical time or display stronger procrastination tendencies. Detailed power-law exponents are listed in Table 3.

Taken together, these findings suggest that the task queue length *L* significantly influences individuals’ temporal participation patterns and responsiveness to critical time effects. Specifically, a large task queue drives enhanced synchronicity in inter-individual task participation timing, but also makes them more prone to long waiting periods and procrastination. In contrast, a small task queue results in more dispersed task initiation times but effectively prevents extreme long-term delays, leading to more stable responsiveness.

#### 3.3.3. The Effects of the Number of Tasks (*N*) Generated at Discrete Time *t*

To investigate the impact of the number of tasks generated at discrete time *i*, denoted by *N*, on the distribution of τ and δ in the model, we conducted simulation experiments while keeping other parameters constant: L=10, C=30, and K=1000. We employed a Barabási-Albert (BA) network as the social network structure, as mentioned earlier. Simulation experiments were conducted with values of *N* equal to 1L, 10L, 100L, and 1000L, respectively. The results are displayed in Figure 3, where varying values of *N* are indicated by distinct colors and shapes.

Figure 3e presents similar results to the previous section, which can also be fitted with an exponential function. The specific results of the fitting parameter analysis are presented in Table 3. It can be observed from the figure that, as the value of *N* increases, the peak of the τ distribution rises significantly and becomes highly concentrated around τ=0, indicating that individuals engage in tasks more synchronously.However, for the same τ value, different values of *N* have little influence on P(τ) on the right side of the peak. In Figure 3f, the δ values for different *N* values all adhere to a power-law distribution, and the power-law exponents exhibit minimal variation across different *N* values.

The numerical simulation results indicate that as *N* increases, it signifies a larger number of tasks in the task repository with deadlines after the current time. At this point, the majority of individuals tend to choose tasks near the deadlines, thereby increasing the likelihood of completing them before their deadlines.

#### 3.3.4. The Effects of the Iteration Step Size (*K*)

To explore the impact of the iteration step size *K* on the distribution of τ and δ in the model, we kept other parameters constant: L=10, C=30, and N=2L, and utilized a Barabási-Albert (BA) network as the social network structure, as described earlier. We conducted simulation experiments for K=250, 500, 1000, and 2000. The results are presented in Figure 3.

In Figure 3g, each color and shape represent a different iteration step size *K*. It is evident that the results exhibit consistency with empirical data and can be fitted with an exponential function. The detailed fitting parameter analysis is provided in Table 3. Notably, on the left side of the peak point, for the same value of τ, smaller *K* values correspond to smaller P(τ), indicating a slower rise. Conversely, on the right side of the peak point, smaller *K* values correspond to larger P(τ), indicating a slower descent. Additionally, as τ values increase at the peak, the frequency of the peak decreases.

Figure 3h displays the distribution of δ for different values of *K*. It can be observed that all values of *K* adhere to a power-law distribution for δ, but the power-law exponents differ. This discrepancy arises because the iteration time directly affects the power-law graph, with smaller *K* values yielding larger power-law exponents.

The numerical simulation results reveal that with an increase in the iteration time, the influence of the neighbor effect on individual task selection relatively increases. As the total time lengthens, an individual’s neighbors are more likely to complete similar tasks, thereby increasing the probability of selecting tasks with a larger δ. This phenomenon weakens the influence of the critical time effect on individual task selection to some extent.

## 4. Conclusions and Discussion

This study significantly advances our understanding of asymmetrical human behavior by integrating task queuing models, social influences, and critical timing effects. By extending task queuing theory and integrating concepts from social influence theory, we provide new insights into the nuanced dynamics of human behavior, particularly in the context of social media messaging behavior.

Our findings underscore the intricate interplay between individuals and their social environments, highlighting the pivotal role of social influence in shaping asymmetrical behavior. Moreover, we demonstrate the substantial impact of critical moments on behavioral asymmetry, emphasizing the dynamic nature of human behavior in response to specific temporal cues. Task queuing theory, as a foundational framework, has been instrumental in our study, continually evolving to account for the multifaceted nature of human behavior and incorporating various intrinsic factors [20,33,34,35].

The empirical analysis conducted in this study, focusing on two distinct time intervals (τ and δ), has yielded promising results. Our numerical simulations, which utilized exponential and power-law distributions to characterize τ and δ, respectively, align closely with actual data. This congruence validates the efficacy of our models in capturing the complexities of social media messaging behavior and accentuates the significance of critical event moments and herd mentality in prompting distinct behavioral patterns.

However, it is important to acknowledge the limitations of our study. Our framework primarily relies on the assumption of “task queuing and preference,” overlooking other intrinsic factors such as human physiology and interest. Second, from a methodological perspective, our modeling of social structures relies on static network models. While effective for simulation, these simplified topologies may not fully capture the temporal evolution, community structure, and dynamic interactions characteristic of real-world social networks. Similarly, the assumption of a fixed task queue length (*L*) serves as a necessary simplification of cognitive constraints; in reality, individual processing capacity may vary dynamically under pressure or specific contexts. Additionally, the abrupt phenomena observed in our empirical data raise questions about potential self-organized criticality mechanisms, warranting further exploration.

Looking ahead, future research should aim to address these limitations and delve deeper into understanding the significance of social media information dissemination. Specifically, future models could incorporate more realistic, dynamic network topologies and adaptive task queue mechanisms to better mimic the complexity of human behavior. Exploring additional potential mechanisms underlying asymmetrical human behavior will further enrich our understanding of this complex phenomenon. Our model serves as a valuable tool for exploring these intricate dynamics and opens up new avenues for research in the field of human behavior analysis.

In summary, our study contributes to a deeper understanding of human behavior dynamics, shedding light on the interplay between social influence, critical timing effects, and task queuing behavior. By elucidating these complex interactions, we pave the way for future research aimed at unraveling the intricacies of human behavior in various contexts.

## Figures and Tables

**Table 1:** The Basic Statistics Information of the Data.

Movie or Event Name	Selected Records Count	Total Records Count	Time Interval (D)	Peak Frequency (P)
Avengers Age of Ultron	51,461	54,153	0	0.09
Big Fish and Begonia	80,529	83,692	1	0.11
Captain America Civil War	60,525	64,410	1	0.13
Forever Young	28,256	30,475	0	0.11
Journey to the West: The Demons Strike Back	82,000	88,903	0	0.20
The Avengers	72,202	78,281	1	0.06
The Continent	110,674	120,200	0	0.11
The Left Ear	36,791	39,802	1	0.05
The Mermaid	68,090	73,882	1	0.07
Tiny Times I	82,000	88,903	1	0.10
Tiny Times III	36,442	41,152	0	0.07
Transformers Age of Extinction	53,958	58,746	1	0.09
Xiaomi SU7 Launch Event	41,750	42,034	0	0.07

**Table 2:** Model Parameters.

Parameter	Description
*G*	Complex network model representing social connections among individuals.
*L*	Length of an individual’s task queue, indicating the number of tasks an individual can hold at a time.
*C*	Time interval between successive task generations.
*N*	Number of tasks introduced to the task repository at each time step.
*K*	Total number of iterations or time steps undergone by the model.

**Table 3:** Parameters for fitting the τ distribution for different numerical results.

Model Parameter	Condition	$a_{1}$	$a_{2}$	$b_{1}$	$b_{2}$	The Power Law Exponent
G	BA	0.13	0.09	0.40	0.16	1.138
	WS	0.13	0.09	0.41	0.15	1.132
L	1	0.07	0.05	0.16	0.09	1.028
	10	0.13	0.09	0.40	0.16	1.138
	100	0.10	0.07	0.55	0.08	1.000
	1000	0.20	0.04	1.50	0.06	1.000
N	1L	0.12	0.09	0.37	0.14	1.114
	10L	0.12	0.09	0.41	0.15	1.136
	100L	0.13	0.08	0.41	0.15	1.135
	1000L	0.24	0.06	1.66	0.09	1.172
K	250	0.11	0.10	0.47	0.14	1.268
	500	0.12	0.09	0.45	0.14	1.162
	1000	0.13	0.09	0.40	0.16	1.137
	2000	0.13	0.09	0.38	0.16	1.111

## Data Availability

The dataset used for this study is partially derived from a publicly available source and partially self-collected. The publicly available portion can be accessed at: https://www.kaggle.com/datasets/utmhikari/doubanmovieshortcomments (accessed on 1 May 2025). The self-collected dataset and the code relevant to this study are available from the corresponding author (zzhidanzhao@gmail.com) upon reasonable request.
